# A Photochemical Avenue to Photoluminescent N-Dots and their Upconversion Cell Imaging

**DOI:** 10.1038/s41598-017-01663-x

**Published:** 2017-05-11

**Authors:** Qingqing Jin, Amu Gubu, Xiuxian Chen, Xinjing Tang

**Affiliations:** State Key Laboratory of Natural and Biomimetic Drugs, Peking University, NO. 38 Xueyuan Road, Beijing, 100191 China

## Abstract

A photochemical avenue to synthesize nitrogen-rich quantum dots (N-dots) using 2-azido imidazole as the starting material was established for the first time. A production yield of up to 92.7% was obtained. The N-dots were then fully characterized by elemental analysis, IR, XPS, XRD, AFM and TEM. On the basis of the N_2_ production and *in situ* IR results, the underlying mechanism for the photochemical formation of N-dots was proposed. These N-dots showed promising optical properties including wavelength-dependent upconversion photoluminescence, and were successfully used in upconversion cell imaging.

## Introduction

Although semiconductive quantum dots have been widely investigated and used in photonics, electronics, and catalysis for many years, their potential long-term toxicity and/or environmental hazards may limit their biological and biomedical applications^1–5^. Therefore, new biocompatible nanomaterials with good optical properties need to be further developed. In 2004, Scrivens serendipitously discovered carbon dots (C-dots) during the preparation of graphene oxide^6^. Sun *et al*. further investigated the optical properties of C-dots with quantum effect^7^. Since then, C-dots have attracted intense attention in many applications such as bioimaging agents^8–13^ and sensors^14–18^ because of their fascinating photoluminescence properties with wavelength-dependent multiple color imaging. In addition, C-dots containing a little amount of nitrogen have been reported to show excellent optical properties^19–21^.

The approaches for synthesizing these non-metal quantum dots are mainly classified into two types: top-down and bottom-up approaches. In the top-down approach, C-dots are prepared from large graphene sheets through chemical or laser ablation, oxygen plasma treatment, electrochemical oxidation, etc. In the bottom-up approach, depending on the starting materials, mainly three methods are used: the acidization of certain carbon-containing materials, carbonization of natural bioresources, and thermal condensation and further carbonization of some organic molecules^22–27^. However, these methods require harsh conditions (large amount strong acids and high temperature, *et al*.). Recently, we serendipitously discovered nitrogen-rich quantum dots (N-dots, up to 34% nitrogen and 10% more nitrogen than carbon) by heating 2-azidoimidazole in methanol or water. These N-dots showed excellent photoluminescence. However, large particles were formed and a yield of around 33% was obtained. In the present work, we demonstrated, for the first time, a photochemical avenue to synthesize N-dots from a small organic compound (2-azidoimidazole) with a large synthetic yield (up to 92.7%). We also proposed the mechanism for the photoinduced formation of N-dots. The photoluminescence properties of the N-dots were investigated and their applications in upconversion photoluminescence cell imaging were also studied.

**Figure 1:**
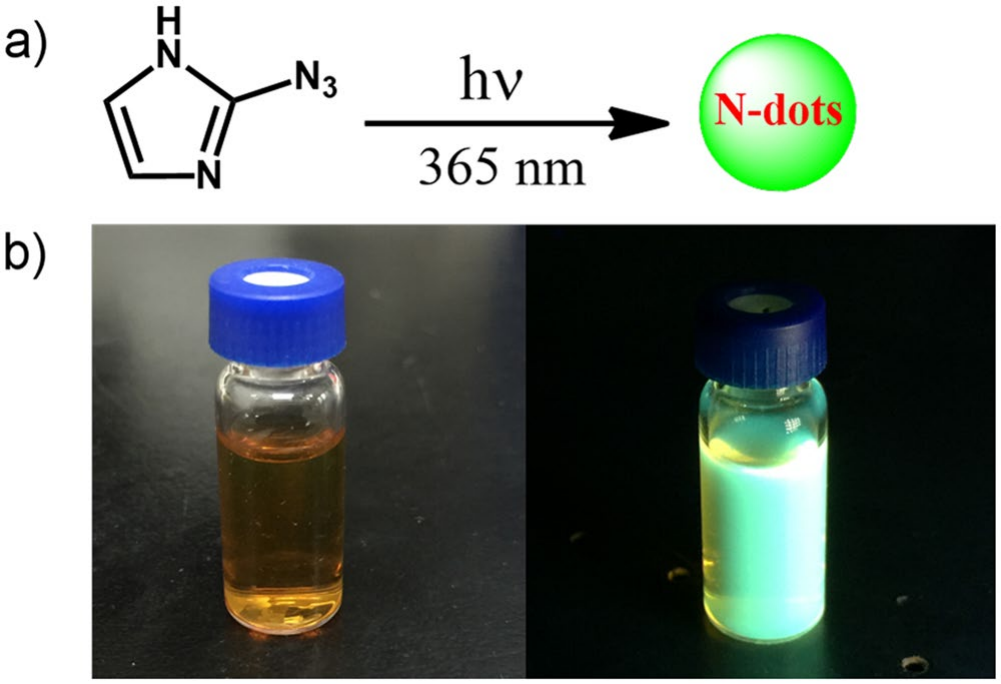
(**a**) Synthesis of N-dots. (**b**) Digital image (left) of 1.0 mg/mL N-dots in water and its photoluminescence image (right) under hand-held UV lamp (365 nm).

**Figure 2:**
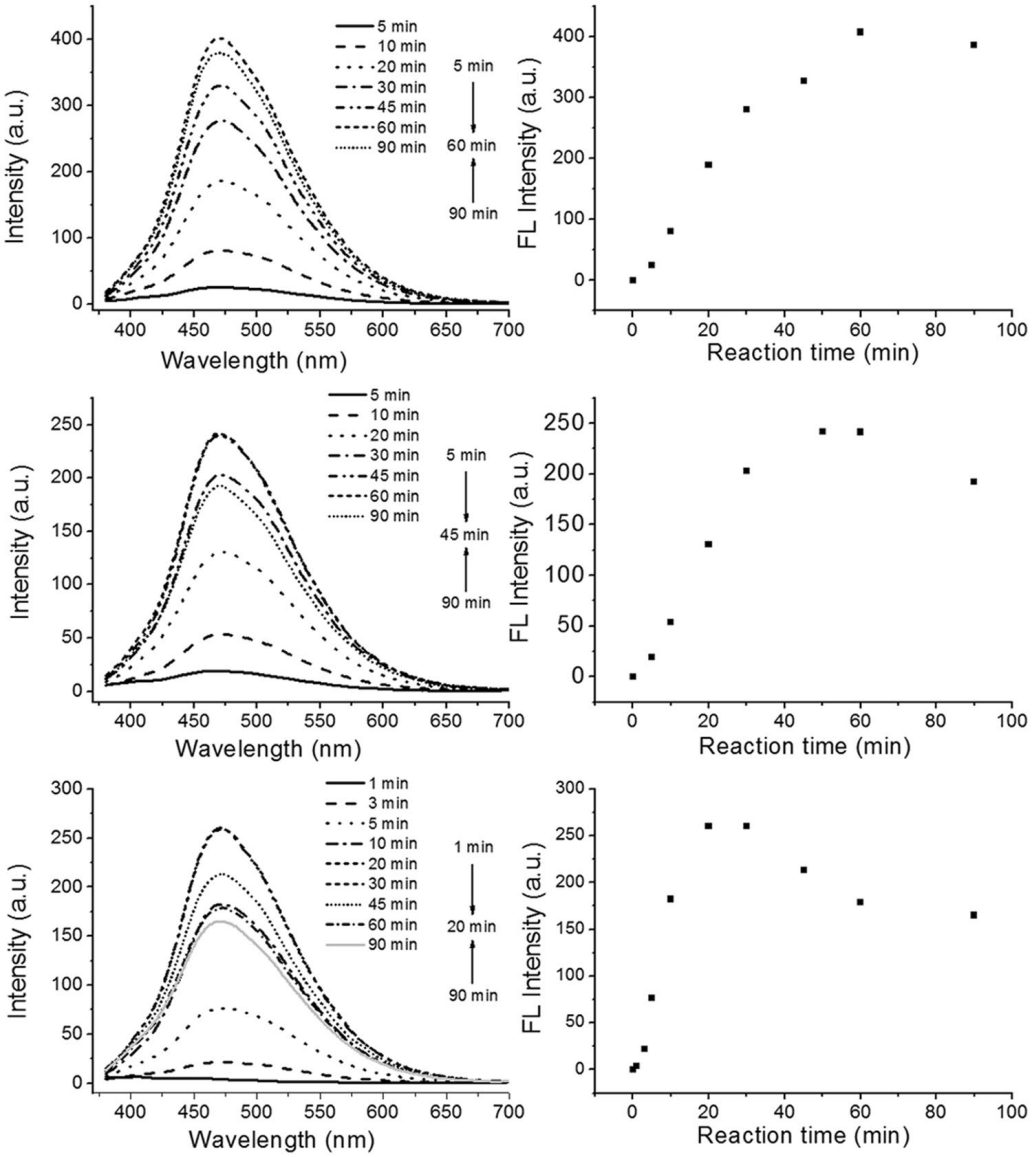
The photoluminescence emission spectra of N-dots produced at different UV-irradiation intensities: (**a**) 20, (**b**) 30, and (**c**) 50 mW/cm^2^.

The N-dots were synthesized by first irradiating a methanol solution of 2-azidoimidazole (Fig. 1). The 2-azidoimidazole solution (5 mg/mL) solution was irradiated with UV light from an LED (365 nm, 50 mW/cm^2^). Upon irradiation, the color of the solution quickly turned from canary to brown and finally to clear dark brown. At the same time, we also observed an intensive cyan-green photoluminescence emission. The ratio of the integral area of photoluminescence intensity to UV absorption at 365 nm increased with the irradiation time and reached a plateau in ~20 min, which marked the completion of the N-dot synthesis. The as-prepared N-dots (Fig. 2) showed the same photoluminescence spectra at varying UV light-irradiation intensities (20, 30, and 50 mW/cm^2^). However, the disappearance of the starting materials was highly dependent on the irradiated UV light intensity. Once the starting materials were fully consumed (around 60, 40, and 20 min for 20, 30, and 50 mW/cm^2^, respectively), the ratios of the integrated photoluminescence intensity to UV absorbance (365 nm) reached the plateau, indicating that the N-dots were fully formed. Further irradiation, especially at high UV light intensity (50 mW/cm^2^) slightly decreased the photoluminescence intensity of the N-dots. However, for longer irradiation with 20 mW/cm^2^ UV light, no obvious decrease in the N-dots photoluminescence was observed.

In our previous work, we prepared N-dots by a thermodynamic method and obtained a muddy solution with the presence of large particles^28^. However, through this photochemical avenue, we obtained a clear brown solution of N-dots. The N-dots could be easily collected by simple filtration and precipitation in ethyl acetate. We obtained the N-dots (0.241 g) with production yield up to 92.7% (calculated using the amount of 2-azido imidazole used i.e., 0.260 g). This yield was much higher than that obtained in our previous study (33%) and that of C-dots prepared by various methods. The elemental analysis results revealed that the percentage of nitrogen in N-dots was up to 35.74%.

The as-synthesized N-dots were highly soluble in water. As shown in Fig. S1a, the N-dots showed a typical UV-Vis absorption spectrum of quantum dots with a broad peak at ~480–530 nm. The photoluminescence spectra shown in Fig. S1b showed that an aqueous solution of N-dots displayed an emission maximum at 480 nm at the excitation wavelength of 360 nm. We also observed a red-shift in the photoluminescence emission peaks (up to 525 nm) with a gradual reduction in their corresponding intensities when the excitation wavelength was increased from 340 to 500 nm in steps of 20 nm. This wavelength-dependent photoluminescence behavior is an indicative of the gradual evolution of confinement in quantum dots. Without any further surface passivation, the photoluminescence quantum yield of the photochemically prepared N-dots was found to be 0.055 at the excitation wavelength of 360 nm using quinine sulfate (0.10 M H_2_SO_4_) as the reference 29. Some studies have reported that the photoluminescence quantum yield of C-dots improves when their surfaces are passivated^7, 19, 30^. A similar trend was expected in the case of our N-dots.

**Figure 3:**
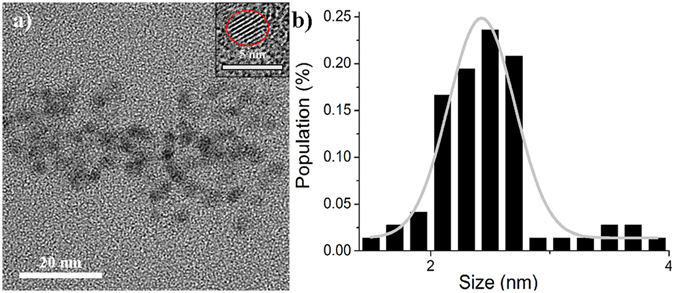
(**a**) TEM image of the N-dots (inset shows the HRTEM image) and (**b**) size distribution histograms of N-dots based on TEM.

The N-dots were characterized by transmission electronic microscopy (TEM), atomic force microscopy (AFM), X-ray diffraction (XRD), Fourier transform infrared (FTIR) spectroscopy, and X-ray photoelectron spectroscopy (XPS). It can be seen clearly from Fig. 3 that the N-dots had a spherical shape and consisted of monodispersed nanoparticles with a diameter of 2.43 ± 0.44 nm. These results agreed well with the height histograms obtained from the AFM measurements (Fig. S2). Furthermore, the typical XRD patterns of the N-dots (Fig. S3) showed that there existed a broad peak centered at 23 °C. The lattice spacing of the core of the N-dots was 0.383 Å, as calculated from Bragg’s equation (2d sin θ = nλ). The XRD broad peak of the N-dots indicated that their surface had an amorphous structure. On the basis of the XRD and high resolution transmission electronic microscopy (HRTEM) results (inset in Fig. 3a), it can be stated that the N-dots had a crystalline internal core with an amorphous surface.

**Figure 4:**
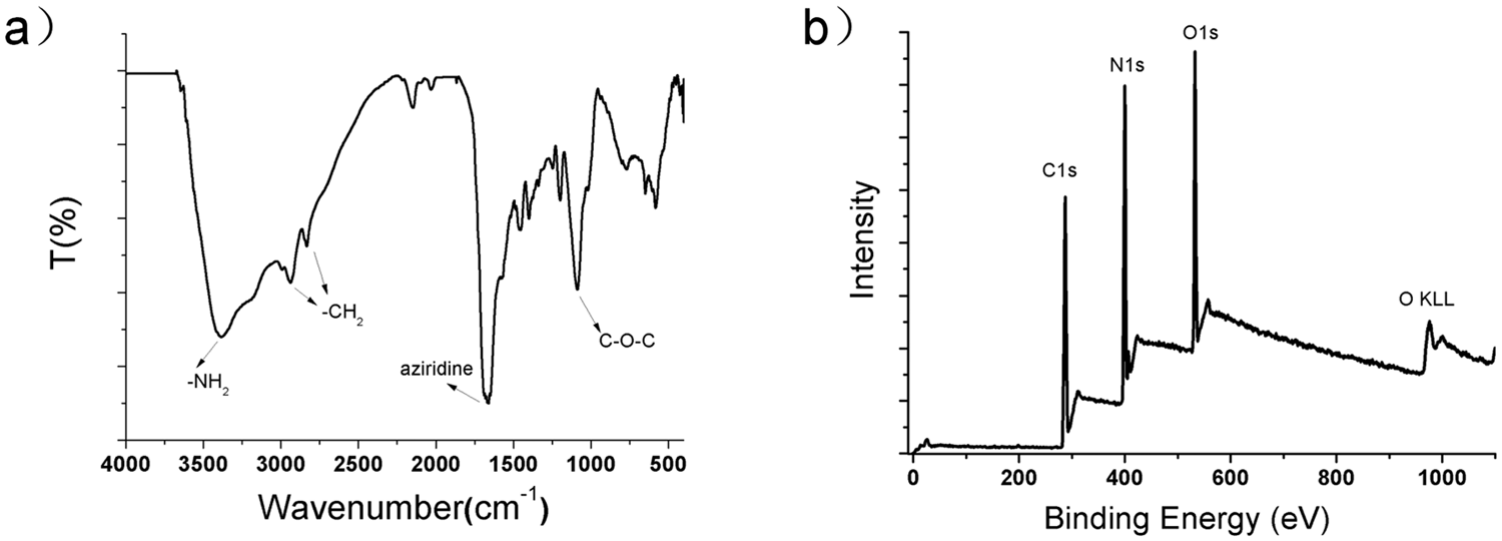
(**a**) FTIR spectrum (at 50 mW/cm^2^) and (**b**) XPS survey spectrum of the photogenerated solid N-dots.

FTIR spectroscopy was used to analyze the fundamental vibrations and properties of the chemical bonds and functional groups of the N-dots. The FTIR spectra of the N-dots (Fig. 4a) revealed that the functional groups such as N–H (3385 and 1544 cm^−1^), C–H (2938 and 2833 cm^−1^), C–O–C (1088 cm^−1^), and aziridine ring (1660 cm^−1^) were present in the N-dots^29, 31–33^. XPS is a surface-sensitive quantitative spectroscopic technique. In this study, it was used to determine the elemental composition, empirical formula, chemical state, and electronic state of the elements on the surface of the N-dots. Fig. 4b shows the typical XPS survey of the N-dots. It can be observed that a high nitrogen content (around 32%) was present on the surface of the N-dots. The XPS results in combination with the elemental analysis results (Table S1) revealed that both the core and surface of the N-dots contained a high percentage of nitrogen and the core contained more nitrogen than the surface did. This can be attributed to the amorphous nature of the surface along with the presence of methoxyl moiety on it. The C1s XPS spectrum of the N-dots (Figs S4–S6 and Table S2) could be deconvoluted into four main components assigned as C–C at ~284.80 eV, C=N at ~286.31 eV, N-C-O at ~287.44 eV, and C–N or C–O at ~288.83 eV^34^. The N1s XPS spectrum of the N-dots also showed three main deconvoluted peaks at around 398.10, 399.85, and 406.42 eV corresponding to the nitrogen atoms of graphitic structure, pyrrolic N, and small amount of NO_2_ groups, respectively^31, 35^. The pyrrolic N was the most abundant (Table S2). This indicates that the N-dots contained a large number of C=N groups. The O1s XPS spectrum of the N-dots could be deconvoluted into peaks at ~531.27 eV, ~532.49 eV and ~534.50 eV, which indicated the presence of oxygen as the form of –OH, C–O and small amount of NO_2_ groups, respectively^31, 36^. Both the FTIR and XPS results showed that the N-dots consisted of a large amount of hydrophilic moieties (such as NH, OH, and C–O). This indicates that the N-dots were highly soluble in water and there was no need for further surface modification and passivation of the quantum dots.

**Figure 5:**
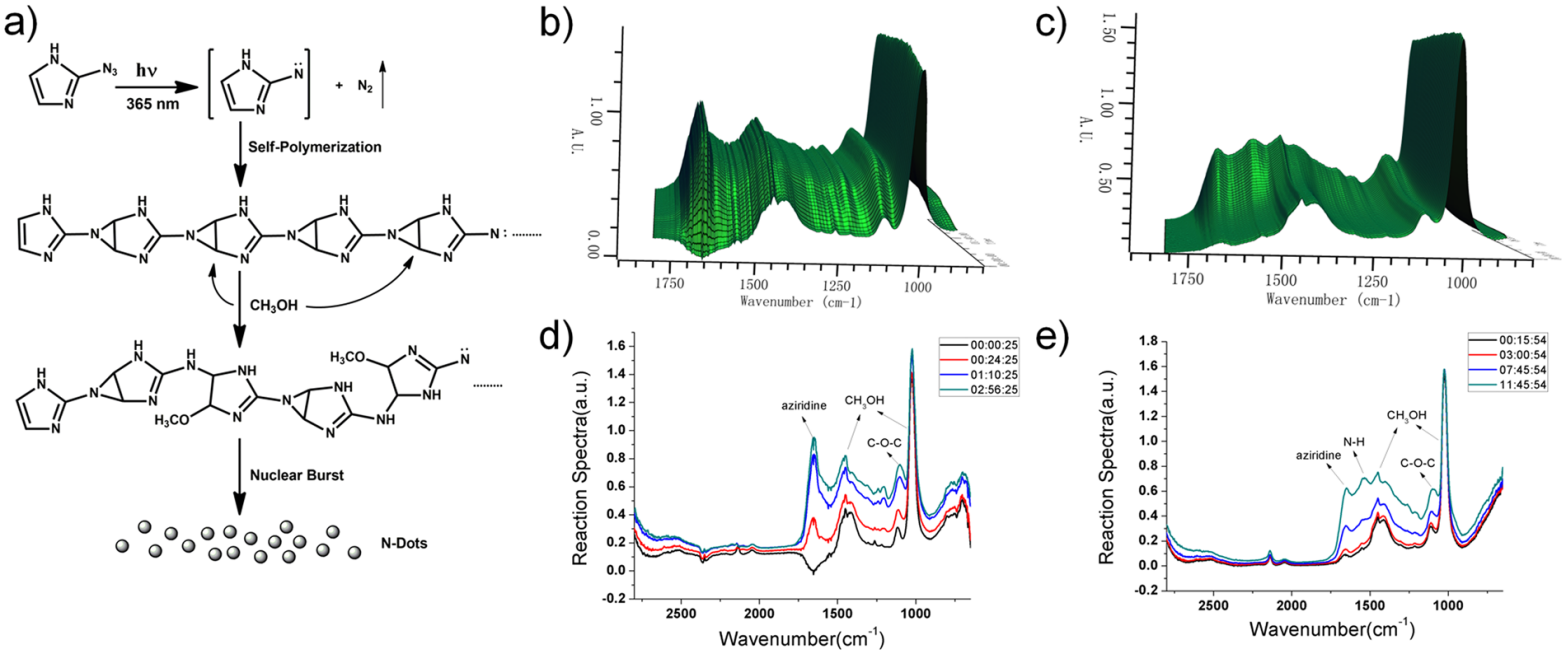
(**a**) Proposed mechanism for the formation of N-dots and *In situ* IR spectra of a 10 mg/mL solution of 2-azido imidazole in methanol in the (**b**) photochemical and (**c**) thermal N-dot formation methods; (**d**,**e**) the typical IR spectra for (**b**) and (**c**), respectively, at different times.

In our previous study, we proposed a mechanism for the thermal preparation of N-dots by trapping a nitrene intermediate with methyl acrylate^28^. In this study also, we tried to use methyl acrylate to trap the photochemically generated active nitrene intermediate. However, a complicated mixture was formed and no trapped product of the active intermediate could be isolated despite the consumption of the starting material. This is possibly because of the occurrence of complicated photochemical reactions (for example, 2 + 2 cycloaddition and photooxidation). To confirm that the active intermediate was generated by the photoinduced decomposition of the azido moiety, the photoinduced nitrogen gas generation from 2-azidoimidazole was monitored (Fig. S7). Once the starting material (2-azidoimidazole) was completely consumed, the total volume of the generated nitrogen gas became equal to the theoretically calculated value (Fig. S7). This confirmed that the active nitrene intermediate was formed by the photoinduced decomposition of the azido moiety. We further investigated the N-dot formation mechanism by comparing the photochemical and thermodynamic methods using *in situ* IR spectroscopy (Fig. 5). As shown in Fig. 5b, a solution of 2-azidoimidazole in methanol showed similar *in situ* IR spectra in both the photochemical and thermodynamic processes. Both spectra showed an increase in the intensity of the peak at 1660 cm^−1^ with time. This peak corresponded to the aziridine ring stretching vibration similar to the trapped product with methyl acrylate (2-aziridinecarboxylic acid-1-(1H-imidazol-2-yl) methyl ester, see supporting Fig. S8). The only difference was that the intensity of the peak at 1544 cm^−1^ (N–H bending vibration) was higher in the thermodynamic method than that in the photochemical method. This is because in the thermodynamic method, the aziridine moieties were attacked by methanol, resulting in the opening of the aziridine rings to form more –NH bonds^28^. In the photochemical method, the N-dots were quickly formed with the dominant unattacked aziridine moieties. On the other hand, the thermal method needed a very long heating time, which resulted in the opening of a large number of aziridine rings. We believe that the photochemical formation of the N-dots started with the photoinduced formation of the active intermediate, followed by the self-polymerization of nitrene and C=C bonds of imidazole with formation of aziridine. Methanol was also involved in N-dots formation by partially opening the ring of aziridine, which was confirmed by HNMR and CNMR (Fig. S9). The N-dots were finally formed after the subsequent nuclear burst of polymers.

**Figure 6:**
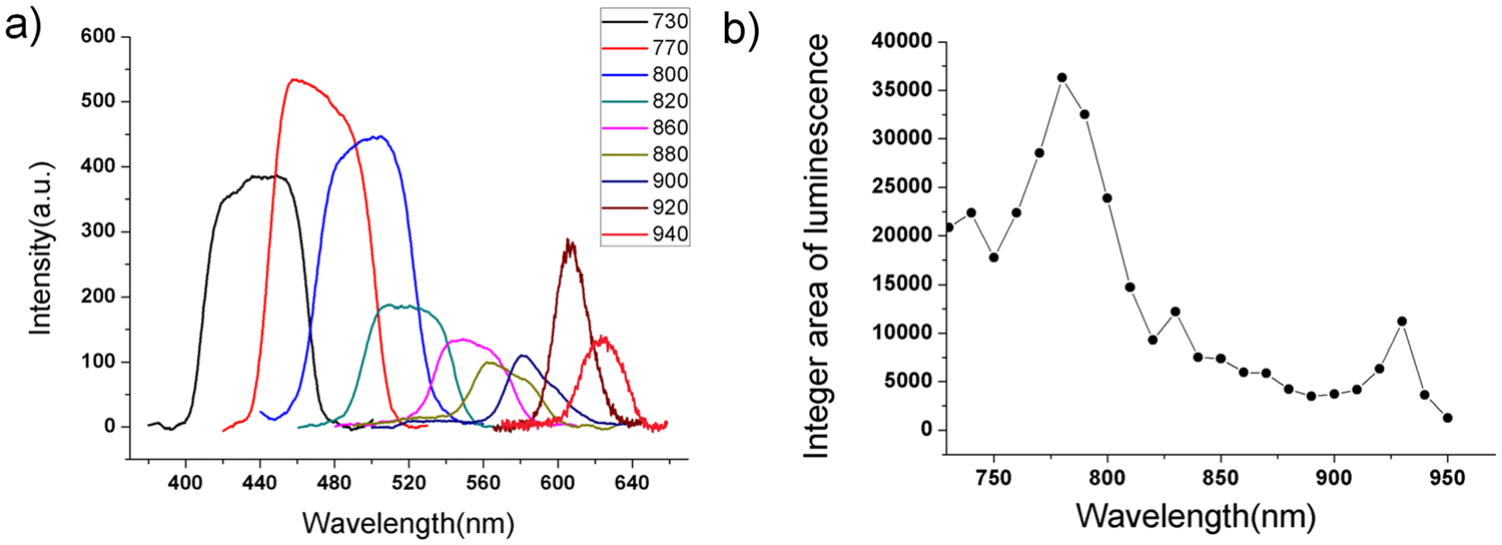
(**a**) Upconversion photoluminescence spectra of an aqueous solution of N-dots at various excitation wavelengths and (**b**) Integration of the area under the photoluminescence peaks at the excitation wavelengths of 730–950 nm in (**a**).

In addition, these N-dots showed strong photoluminescence emissions when excited by a near-IR femtosecond-pulsed laser. The upconversion photoluminescence spectra of the N-dots were obtained at excitation wavelengths in the range of 730–950 nm. The wavelength-dependent upconversion photoluminescence behavior of these N-dots was also clearly observed. Their emission wavelength red-shifted with an increase in the excitation wavelength of the IR laser (Fig. 6). When the N-dots were excited by a laser wavelength from 770 to 900 nm, a very broad upconversion luminescence was observed in the range of 400–580 nm, as shown in Fig. 6a. Interestingly, the N-dots showed a red-shift in the photoluminescence emission (up to 620 nm) with a little bit sharper peaks upon excitation with pulsed laser wavelengths greater than 900 nm. This phenomenon is similar to the observation of single photon excitations due to the gradual evolution of the confinement in quantum dots. The areas under the upconversion photoluminescence peaks at different excitation wavelengths were integrated. The integral intensity was then plotted as a function of the excitation wavelength. The resulting graph (Fig. 6b) showed two broad peaks at around 770 and 920 nm. However, the laser power-dependence of the luminescence intensity did not fit the proposed quadratic relationship of two-photon luminescence (Fig. S11), which is inconsistent with the results obtained for C-dots^8^. Instead, these N-dots exhibited an upconversion luminescence similar to that shown by upconversion nanomaterials^37, 38^.

**Figure 7:**
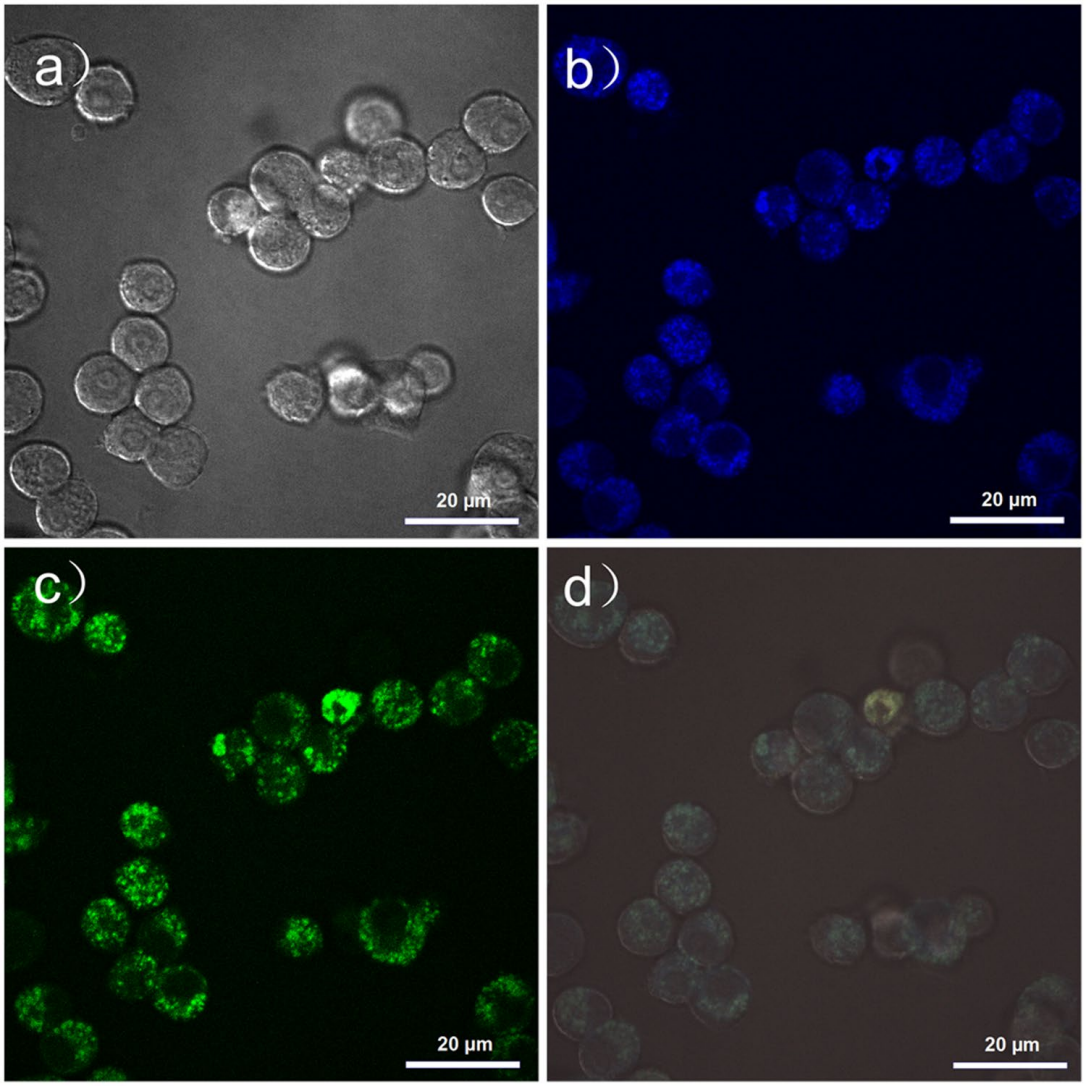
Representative upconversion photoluminescence images of RAW 264.7 cells incubated with N-dots (0.5 mg/mL) for 6 h at 37 °C. (**a**) Bright field image, (**b**,**c**) photoluminescence images obtained at (**b**) 425–475 nm and (**c**) 500–550 nm under 770 nm excitation, (**d**) superimposed image of (**b**) and (**c**). Scale bar: 20 µm.

The upconversion photoluminescence properties of these N-dots were then used for upconversion cell imaging by IR laser excitation with a multiphoton confocal microscope (Fig. 7). The cell viability was first evaluated. As shown in Fig. S12, the N-dots showed low toxicity at the concentration up to 1000 μg/mL. After their incubation with RAW 264.7 cells, the N-dots showed a strong upconversion photoluminescence in blue (425–475 nm) and green (500–550 nm) channels when excited by a wavelength of 770 nm, as shown in Fig. 7b–d. The superimposed image indicates that the photoluminescence in both the channels came from the same N-dot nanoparticles (Fig. 7d). In addition, the upconversion photoluminescence images also suggested that the N-dots were mostly located in the cell cytoplasm instead of the nucleus. This is similar to the cellular distribution of C-dots.

In conclusion, we developed a novel photochemical avenue for the synthesis of a new class of N-dots using 2-azidoimidazole as the starting material. The N-dots could be easily collected and a yield of about 92.7% was obtained. This yield is much higher than those reported for C-dots and for the N-dots prepared by thermal method. The resulting N-dots were fully characterized by elemental analysis, XPS, XRD, FTIR, TEM, and AFM. Their optical properties were also investigated. In addition, we believe that the photoinduced N-dot formation started with the photogeneration of the active nitrene intermediate followed by the nitrene-mediated self-polymerization and condensation. Moreover, the N-dots exhibited a strong photoluminescence emission upon IR laser excitation and showed similar wavelength-dependent photoluminescence properties. However, the IR laser-induced photoluminescence emission of the N-dots was not due to two-photon absorption. Instead, they exhibited an upconversion photoluminescence. The upconversion photoluminescence of the N-dots was successfully used for upconversion photoluminescence cell imaging. The results showed that N-dots are promising nanoprobes for cell imaging and targeting after further surface modification.

## Electronic supplementary material

N-dots-SI-scirep-20170317
