# Supplementary material for: A Photochemical Avenue to Photoluminescent N-Dots and their Upconversion Cell Imaging
Source: Sci Rep. 2017 May 11;7:1793. doi: 10.1038/s41598-017-01663-x (PMC5431983; doi:10.1038/s41598-017-01663-x)
Supplement: Supplementary file 1 — N-dots-SI-scirep-20170317 [file 41598_2017_1663_MOESM1_ESM.pdf]

# Supporting information

# A Photochemical Avenue to Photoluminescent N-Dots and their Upconversion Cell Imaging

Qingqing Jin, Amu Gubu, Xiuxian Chen and Xinjing Tang*

State Key Laboratory of Natural and Biomimetic Drugs, School of Pharmaceutical Sciences, Peking University, No. 38 Xueyuan Rd. Beijing 100191, China.

* Tel: +86-010-82805635; Fax: +86-010-82805635; Email: xinjingt@bjmu.edu.cn

# Experimental Methods

2-aminoimidazole sulfuric acid salt (Ouhe company) was used as received. All the chemicals and reagents were of analytical grade and used without further purification. The deionized water was used in all experiments.

**Synthesis of 2-Azidoimidazole**

2-Azidoimidazole was prepared by the reaction of 2-aminoimidazole sulfuric acid salt with $NaNO_2$/HCl, followed by the addition of$NaN_3$, according to the method described by Chen[1].

**The photochemical preparation of N-dots**

The photochemical preparation of nitrogen-rich quantum dots (N-dots) was performed with methanol solution of 2-azidoimidazole. 2-azidoimidazole (0.260 g) in methanol (20 mL) was irradiated with LED UV light (365 nm, 20, 30 and 50 mW/cm$^2$) until the disappearance of starting materials. After the reaction was complete, the clear methanol solution was concentrated under reduced pressure. The obtained residue was washed by ethyl acetate for 3 times and dispersed in pure water (10 mL). After simple filtration, the supernatant was concentrated and vacuum-frozen-dried, yielding 0.241 g solid, 92.7% yield.

**Characterizations of N-dots**

$^1$H NMR (400 MHz), and $^{13}$C NMR (101 MHz) spectra were recorded on a Bruker spectrometer. Transmission electron microscopy (TEM) and high-resolution transmission electron (HRTEM) images were taken on a Field-emission High Resolution Transmission Electron Microscope (JEM-2100F), using an accelerating voltage of 200 kV. The measurement of C, H, and N elements were performed on Varo EL III elemental analyzer. X-ray photoelectron spectroscopy (XPS) measurements were carried out with an Axis Ultra X-ray photoelectron spectrometer (Kratos Analytical Ltd) with an exciting source of Al Ka, hv=1486.7 eV. Infrared spectra were recorded by

using a NEXUS-470 Fourier transform infrared (FTIR) spectrometer in KBr media. UV-visible (UV-vis) absorption spectra were recorded on DU-800 (Beckman). The photoluminescence (FL) spectra and quantum yields (QY) were measured on a fluorometer (Cary Eclipse) equipped with an integrating sphere. Upconversion photoluminescence imaging of cells was performed in a multiphoton confocal microscope (Nikon A1R MP).

**Mechanism of N-dots formation**

The volume of nitrogen gas produced in photochemical reaction was measured by drainage method. 2-azidoimidazole (36 mg, 0.33 mmol) in methanol (4 ml) was irradiated with LED UV light for 60 min. 7.1 ml nitrogen was obtained finally in ~ 15 min and no further nitrogen production was observed even for long irradiation (up to 45 min). The total volume of generated $N_2$ was consistent to the theoretically calculated value (Figure S8), which confirmed nitrene was formed after the decomposition of azido moiety.

Furthermore, we compared the FTIR spectra of 2-azidoimidazole and trapped product of active intermediate (2-aziridinecarboxylic acid-1-(1H-imidazol-2-yl) methyl ester) in thermodynamic method, and found that peak at ~ 1660 $cm^{-1}$ was newly generated in the captured product, indicating the aziridine ring stretching vibration (Figure S9). In addition, peak at 1660 $cm^{-1}$ in the FTIR spectra of photochemically produced N-dots was obvious, which confirmed a large amount of aziridine rings in N-dots.

We further monitored the reaction progress of N-dots formation by comparing photochemical and thermodynamic methods using *in situ* IR spectroscopy. 2-azidoimidizole methanol solution (10 mg/ml) dissolved in quartz flask was irradiated by LED UV lamp for 4 h, while the same solution (10 mg/ml) was heated at 60 $^{o}$C for 12 h. *In situ* IR spectra obtained from the two reactions were similar, displaying 1654 $cm^{-1}$ (aziridine ring stretching vibration), 1450 $cm^{-1}$(C-H bending vibration from $CH_3OH$ solvent), 1085 $cm^{-1}$ (C–O–C stretching vibration), 1025 $cm^{-1}$ (C–O stretching vibration from $CH_3OH$ solvent). However, 1544 $cm^{-1}$ (N-H bending vibration due to

the opening of aziridine ring) was much more obvious in thermodynamic than photochemical preparation method.

## Measurement of the photoluminescence quantum yield

The absolute quantum yield value was measured using the following equation:

$$\Phi_{s} = \Phi_{r}\left[\frac{A_{r}(\lambda_{r})}{A_{s}(\lambda_{s})}\right]\left[\frac{I_{r}(\lambda_{r})}{I_{s}(\lambda_{s})}\right]\left[\frac{n_{s}}{n_{r}}\right]^{2}\left[\frac{F_{s}}{F_{r}}\right]$$

Where Φ is the quantum yield; A($\lambda$) is absorbance and $I(\lambda)$ is the relative intensity of exciting light at wavelength $\lambda$; n is refractive index of the solvent and F is the integrated area under the emission spectrum; The subscripts "s" and "r" refer to the unknown quantum yield of N-dots and standard quantum yield of reference quinine sulfate, respectively. The quinine sulfate (literature quantum yield 0.54 at 360 nm) was dissolved in 0.1 M $H_2SO_4$ (n = 1.33) and the N-dots were dissolved in distilled water (n = 1.33). Absorbance in the 1 cm photoluminescence cuvette was kept under 0.05 at the excitation wavelength of 360 nm.

## Measurement of the two-photon absorption cross-section

The two-photon absorption cross-section ($\delta_{TPA}$) was measured by using the two-photon induced photoluminescence method and calculated using the following equation:

$$\delta_{s} = \delta_{r}\frac{C_{r}}{C_{s}}\frac{n_{r}}{n_{s}}\frac{F_{s}}{F_{r}}\frac{\Phi_{r}}{\Phi_{s}}$$

where δ is the TPA cross-section, C and n are the concentration and refractive index of the sample solution, and F is the integrated area obtained from two-photon photoluminescence spectra. Subscripts "s" and "r" refer to the unknown TPA cross-section of N-dots and standard TPA cross-section of fluorescein as reference, respectively. The fluorescein was dissolved in NaOH aqueous solution (pH = 13) at a concentration of $1.0\times10^{-4}$ M and N-dots were dissolved in distilled water at a concentration of 1.0 mg/ml. Two-photon photoluminescence spectra were recorded using an SD2000 spectrometer (Ocean Optics), excited by a femtosecond laser (Mai Tai, Spectra-Physics, Fremont, CA) with a pulse width of 100 fs and a repetition rate of

80 MHz in the wavelength range of 730–870 nm.

The two-photon photoluminescence was confirmed by the dependence of observed luminescence intensities on the excitation laser power. In theory, the absorption of sample is a TPA process under the condition that the slope (K) of the relationship between the logarithm of the photoluminescence integral area (F) and the laser energy (P) of sample is around 2. The two-photon absorption (TPA) nonlinear relationship of reference fluorescein and N-dots were shown in Figure S11. The slope of fluorescein and N-dots was 2.01 and 0.69 at the wavelength of 800 nm, indicating that no quadratic relationship between the excitation laser power and the luminescence intensity of N-dots solution was observed.

**Cell culture and cellular viability analysis**

Macrophage cell line, RAW 264.7 was grown in DMEM containing 10% heat-inactivated fetal bovine serum in an atmosphere of 5% $CO_2$, 37 ℃. To evaluate the toxicity of N-dots, macrophages were incubated with varying concentrations (0–1000 μg/mL) of N-dots for 24 h and then analyzed with standard sulforhodamine B assay. As seen in Figure S12, no significant cytotoxic effect was observed with 0.5 mg/ml N-dots when upconversion photoluminescence imaging.

**Upconversion photoluminescence imaging of N-dots in cells**

RAW 264.7 cells were plated at $5 \times 10^5$ cells per well in cell culture dishes for 24 h. A solution of the N-dots (5 mg/mL) in PBS buffer was passed through a 0.2 μm sterile filter membrane. The filtered solution (100 μL) was mixed with DMEM (900 μL) and then added to each well and incubated for 6 h. After washing with PBS for two times, fresh DMEM was added to the plate for optical imaging. Upconversion photoluminescence imaging of RAW264.7 cells was performed in a confocal microscope, and the photoluminescence images of cells were captured.

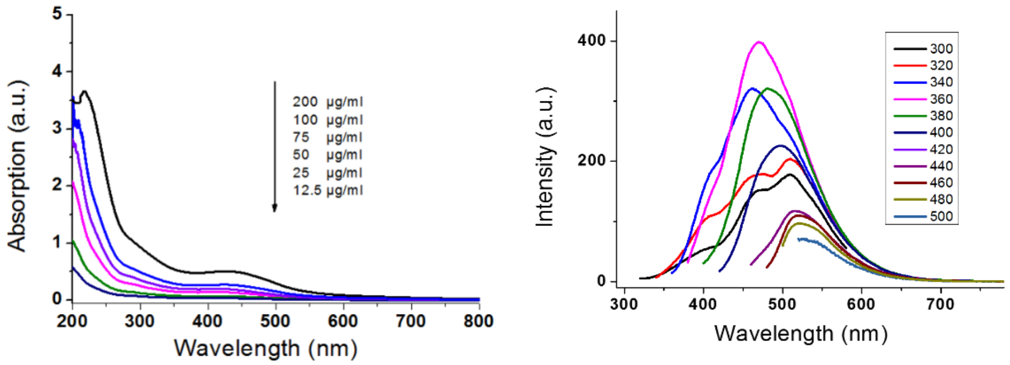


***Figure S1.*** UV-vis spectra of different concentrations of N-dots solutions, and the photoluminescence emission spectra of N-dots solution (200 µg/mL, recorded from 300 to 500 nm in 20 nm increments).

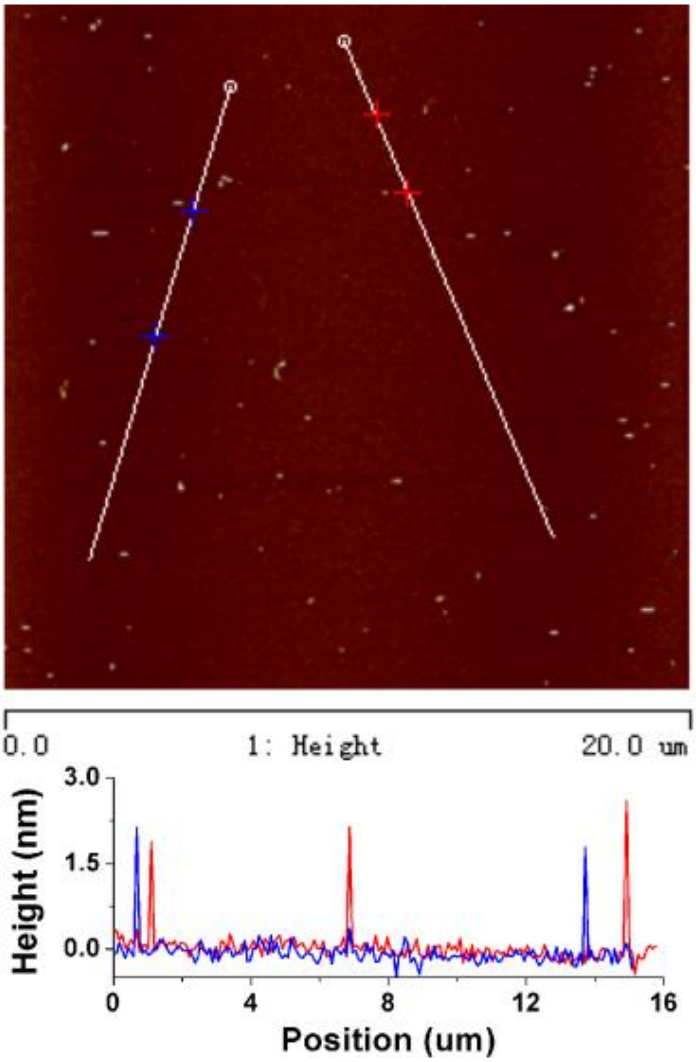


***Figure S2.*** AFM images and size distributions of photochemically synthesized N-dots. Height profiles are given for the marked white line in the AFM image.

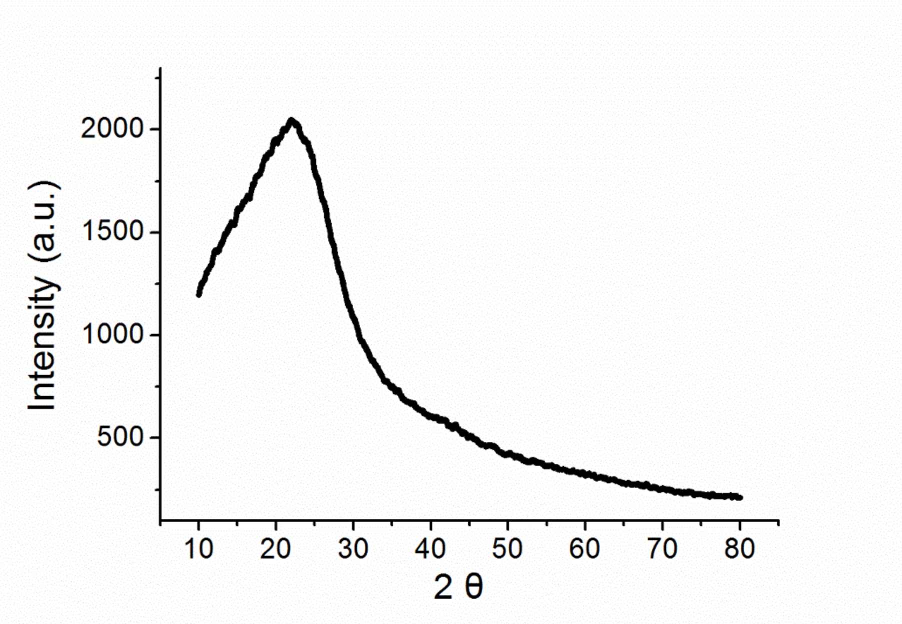


***Figure S3.*** XRD of photochemically synthesized N-dots, showing the broad peak at $2\theta \sim 23°$, d=0.386 nm

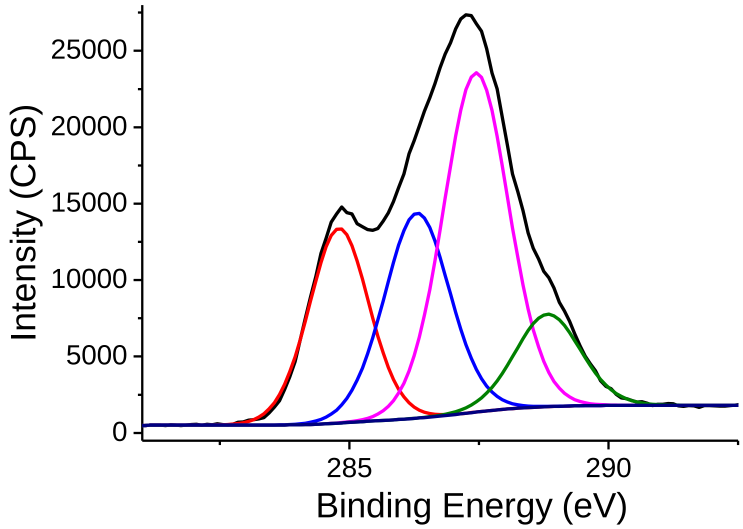


***Figure S4.*** The deconvoluted C1s spectra of N-dots（XPS survey）

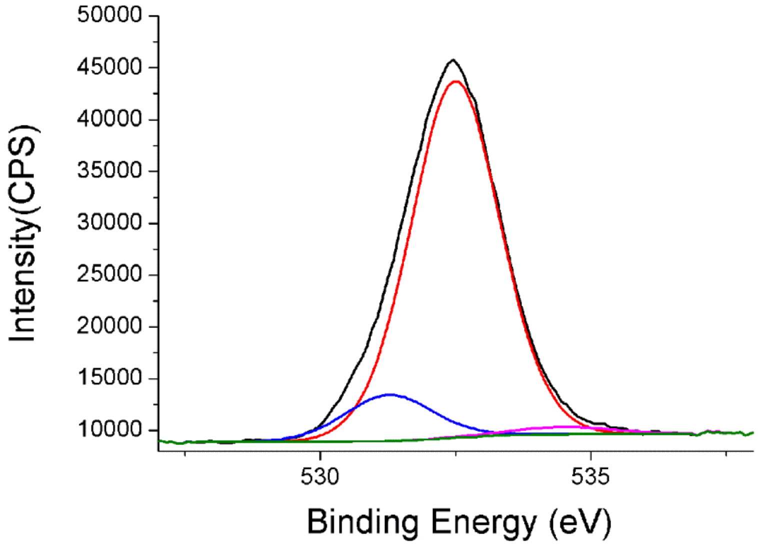


***Figure S5.*** The deconvoluted O1s spectra of N-dots（XPS survey）

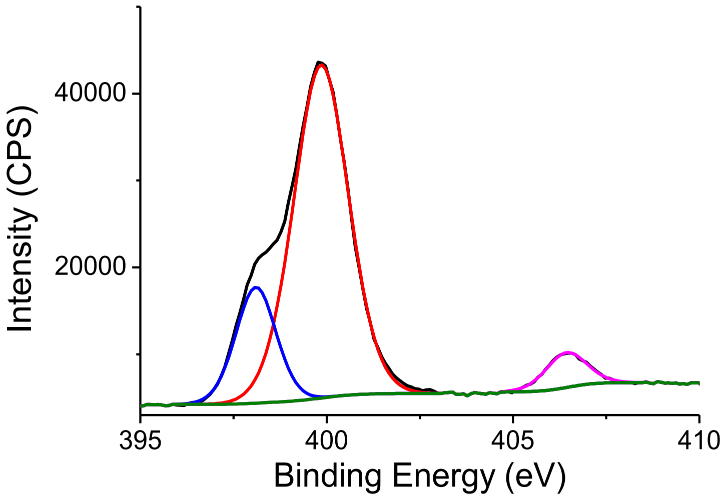


***Figure S6.*** The deconvoluted N1s spectra of N-dots（XPS survey）

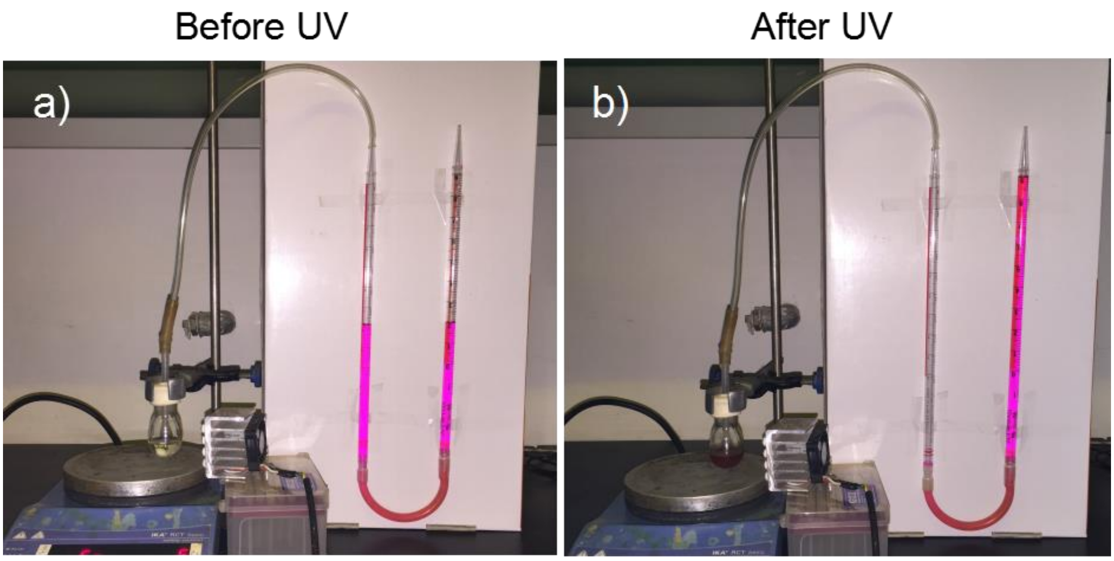


***Figure S7.*** Digital image of the device for monitoring nitrogen gas volume a) before and b) after 15 min UV irradiation.

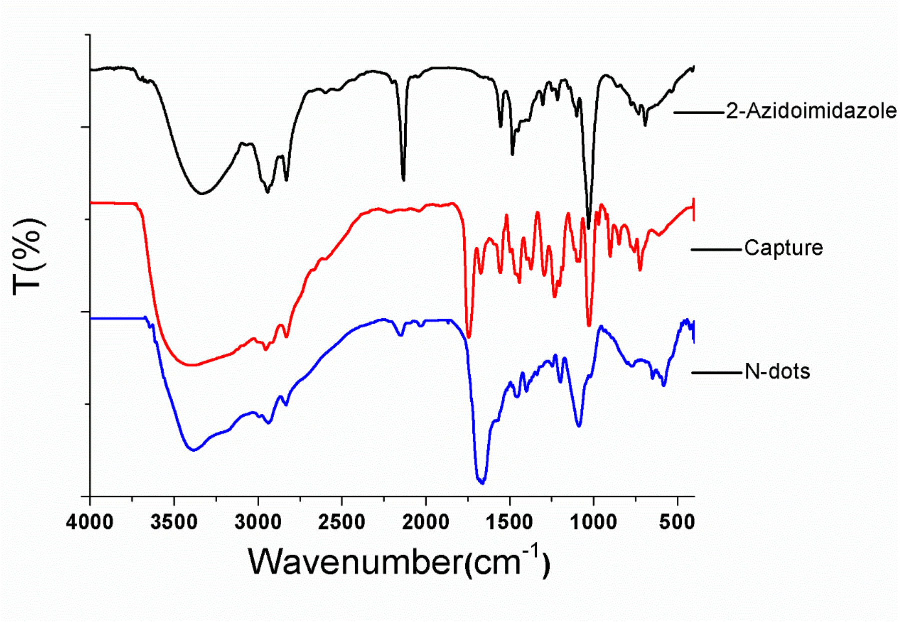


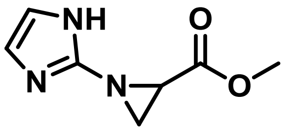


**Capture**

(2-aziridinecarboxylic acid-1-(1H-imidazol-2-yl) methyl ester

***Figure S8.*** FTIR spectra of 2-Azidoimidazole, capture (2-aziridinecarboxylic acid-1-(1H-imidazol-2-yl) methyl ester and photochemically synthesized N-dots produced at 365 nm, 50 mW/cm$^2$.

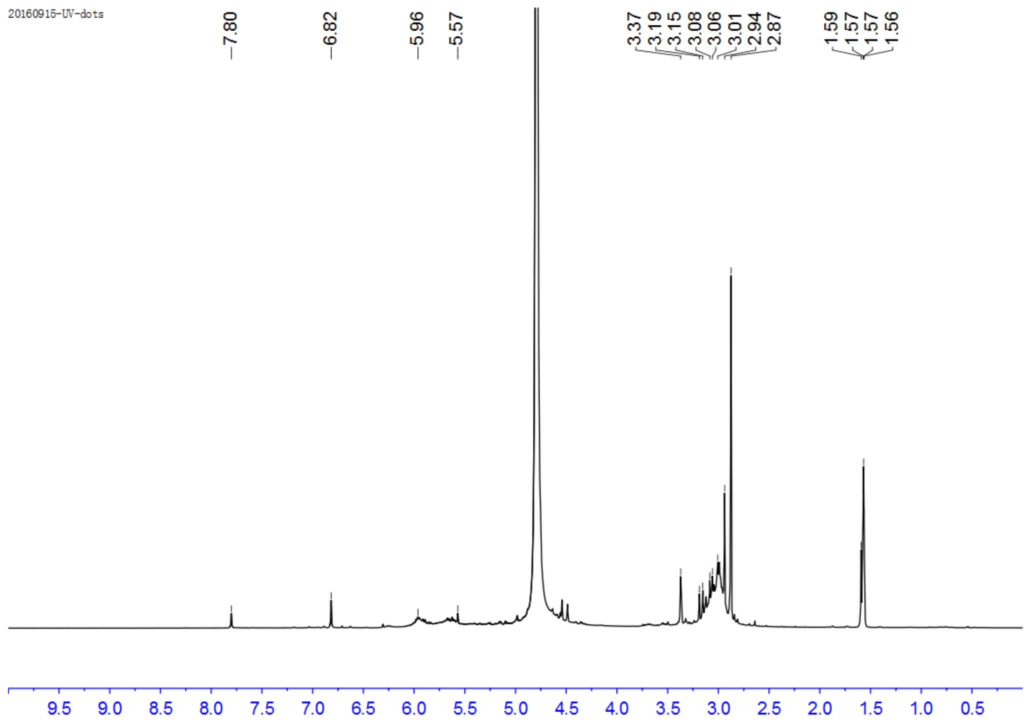


$^{1}$HNMR of N-dots (400 MHz, $D_2O$, $CD_3COOD$) d (ppm): δ 7.80 (s), 6.82 (s), 4.79 (s, $H_2O$), 3.37 (s), 2.87-3.19 (m), 1.56-1.59 (m)

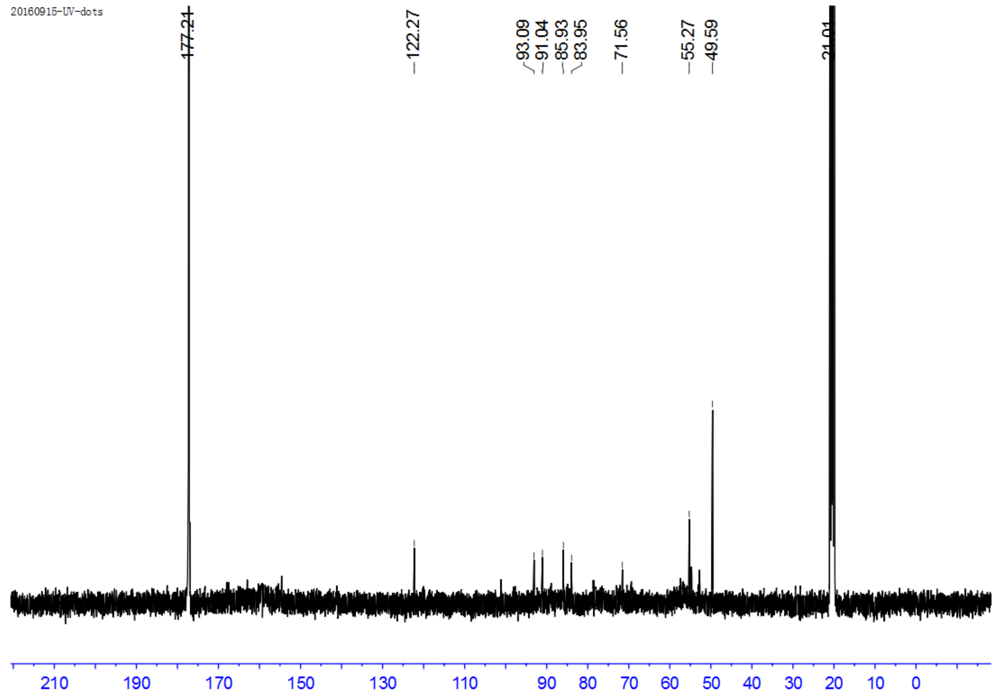


$^{13}$CNMR of N-dots (400 MHz, $D_2O$, $CD_3COOD$) d (ppm): δ 177.21, 122.21, 93.09, 91.04, 85.93, 83.95, 71.56, 55.27, 49.59, 21.01

***Figure S9.*** $^{1}$H-NMR and $^{13}$C-NMR of photochemically synthesized N-dots.

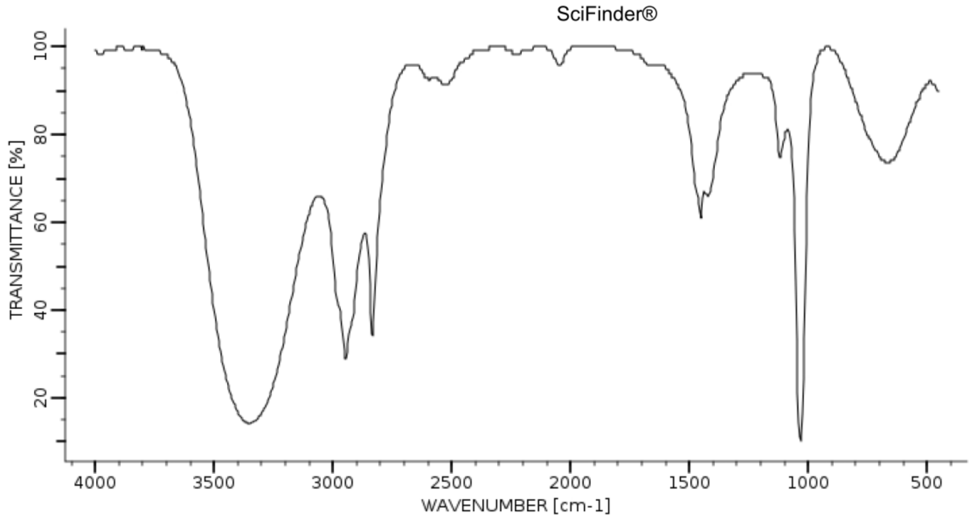


***Figure S10.*** FTIR spectra of methanol (The spectral data was obtained from Bio-Rad/Sadtler IR Data Collection).

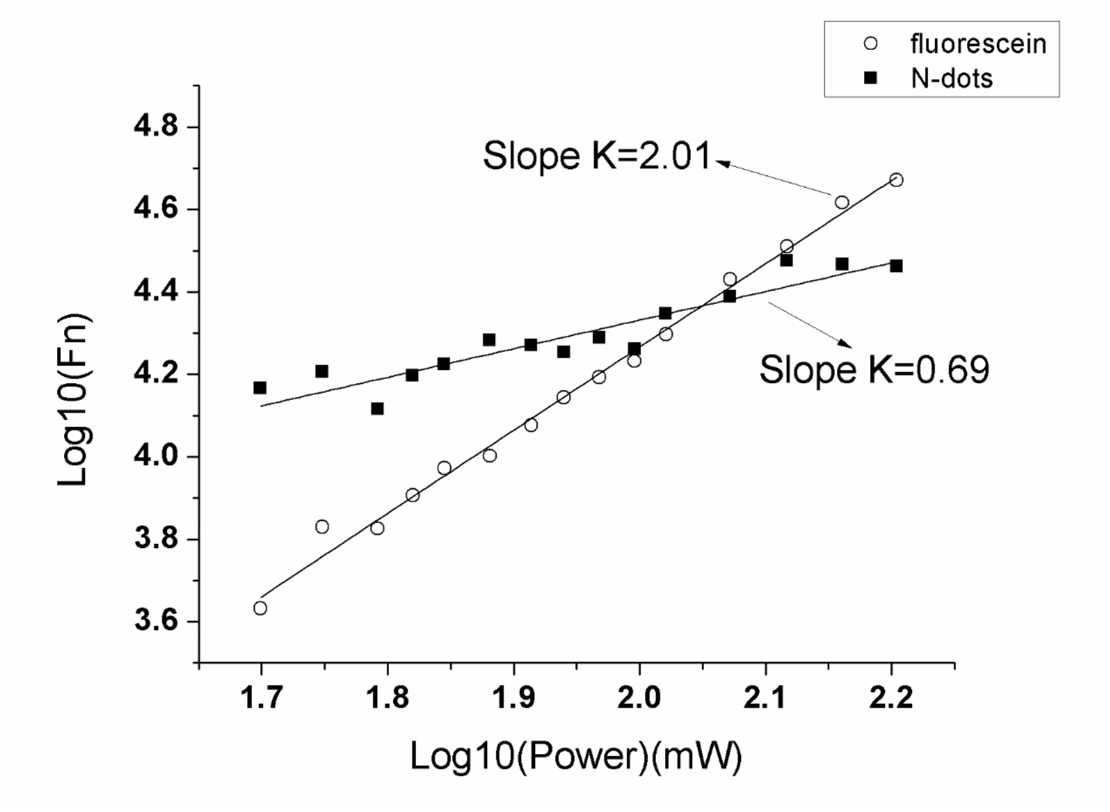


***Figure S11.*** The relationship between two-photon photoluminescence and the laser power of fluorescein and N-dots solutions.

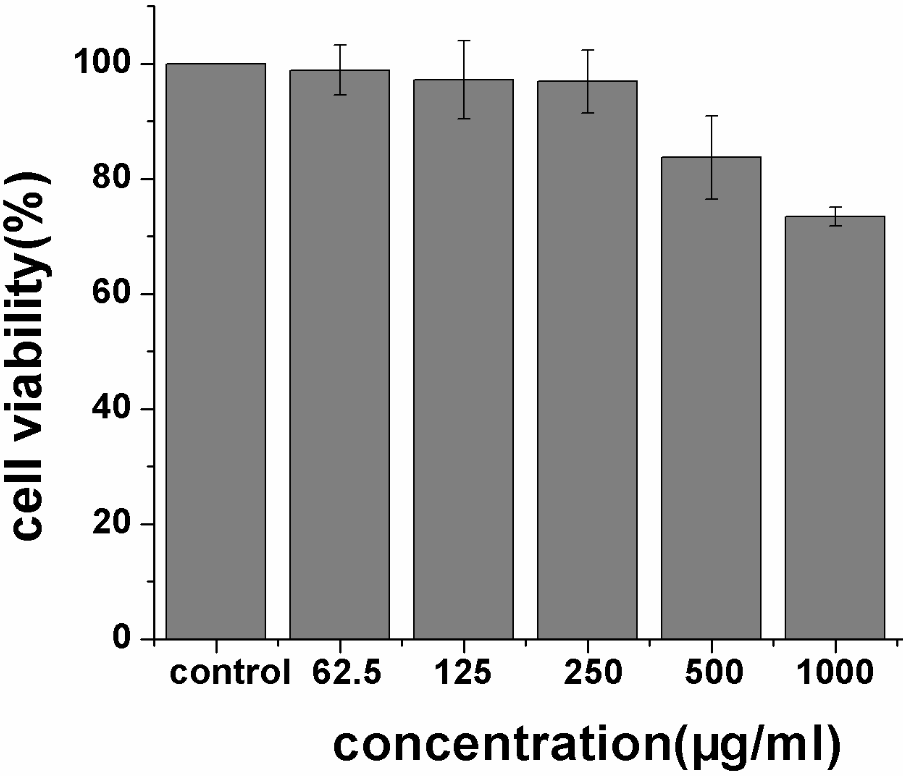


***Figure S12.*** Cellular viability analysis of photochemically synthesized N-dots.

***Table S1.*** Element composition percentage of N-dots (50 mW/cm$^2$)

| Element analysis | Composition percentage (%) of N-dots |
|---|---|
| C | 38.34 |
| H | 5.75 |
| N | 35.74 |

***Table S2.*** Raw date and the deconvoluted C1s, N1s and O1s data of N-dots, and the relative contents of different C, N, O atoms in the N-dots (XPS survey)

| Binding energy (ev) | | Area (%) |
|---|---|---|
| Raw data of N-dots | 287.57(C1s) | 50.84 |
| | 399.57(N1s) | 32.07 |
| | 532.57(O1s) | 17.09 |
| Deconvoluted C1s | 284.80 | 21.91 |
| | 286.31 | 25.17 |
| | 287.44 | 41.60 |
| | 288.83 | 11.33 |
| Deconvoluted N1s | 398.10 | 19.20 |
| | 399.85 | 75.01 |
| | 406.42 | 5.79 |
| Deconvoluted O1s | 531.27 | 11.08 |
| | 532.49 | 86.86 |
| | 534.50 | 2.07 |

1 Chen, X. X., Jin, Q. Q., Wu, L. Z., Tung, C. H. & Tang, X. J. Synthesis and Unique Photoluminescence Properties of Nitrogen-Rich Quantum Dots and Their Applications. *Angew. Chem. Int. Ed.* **53**, 12542-12547, doi:10.1002/anie.201408422 (2014).
